# Pharmacological Utility of PPAR Modulation for Angiogenesis in Cardiovascular Disease

**DOI:** 10.3390/ijms24032345

**Published:** 2023-01-25

**Authors:** Nicole Wagner, Kay-Dietrich Wagner

**Affiliations:** CNRS, INSERM, Université Côte d’Azur, iBV, 06107 Nice, France

**Keywords:** peroxisome proliferator activated receptors, cardiovascular disease, angiogenesis, myocardial infarction, clinical trials, aspirin, statins, angiotensin-converting enzyme inhibitors, angiotensin receptor blockers

## Abstract

Peroxisome proliferator activated receptors, including PPARα, PPARβ/δ, and PPARγ, are ligand-activated transcription factors belonging to the nuclear receptor superfamily. They play important roles in glucose and lipid metabolism and are also supposed to reduce inflammation and atherosclerosis. All PPARs are involved in angiogenesis, a process critically involved in cardiovascular pathology. Synthetic specific agonists exist for all PPARs. PPARα agonists (fibrates) are used to treat dyslipidemia by decreasing triglyceride and increasing high-density lipoprotein (HDL) levels. PPARγ agonists (thiazolidinediones) are used to treat Type 2 diabetes mellitus by improving insulin sensitivity. PPARα/γ (dual) agonists are supposed to treat both pathological conditions at once. In contrast, PPARβ/δ agonists are not in clinical use. Although activators of PPARs were initially considered to have favorable effects on the risk factors for cardiovascular disease, their cardiovascular safety is controversial. Here, we discuss the implications of PPARs in vascular biology regarding cardiac pathology and focus on the outcomes of clinical studies evaluating their benefits in cardiovascular diseases.

## 1. Introduction

Peroxisome Proliferator Activated Receptors are ligand-activated transcription factors regulating energy homeostasis. They include PPARα, PPARβ/δ, and PPARγ. PPARα is highly expressed in skeletal muscle, the heart, the liver, and brown adipose tissue and acts as a major regulator of fatty acid oxidation (FAO), thereby lowering lipid levels. PPARβ/δ is ubiquitously expressed and involved in lipid catabolism, inflammation, and energy dissipation. PPARγ expression is highest in adipose tissue, the large intestine, and the spleen, where it regulates adipogenesis, lipid and glucose metabolism, and inflammatory pathways. Thus, PPARs coordinate a variety of metabolic pathways upon activation by endogenous ligands, mainly produced in the metabolic pathways of fatty acids or synthetic modulators. After interaction with the ligands, PPARs are translocated to the nucleus, where they change their structure and regulate gene transcription. PPAR agonists have variable properties and specificities for individual PPAR receptors, different absorption and distribution profiles, and differing gene expression profiles, which accordingly lead to distinct clinical outcomes. PPARα agonists (fibrates) are used to treat dyslipidemia by decreasing triglyceride and increasing high-density lipoprotein (HDL) levels (reviewed in [1]). The PPARβ/δ agonist GW501516 entered clinical development as a drug candidate for cardiovascular and metabolic diseases, but clinical trials had to be stopped due to rapid cancer appearance observed in animal studies [2,3]. PPARγ agonists (thiazolidinediones) improve insulin sensitivity and are used in the therapy of Type 2 diabetes [1]. The effectiveness of these PPAR activators in the treatment of metabolic disorders, such as hyperlipidemia and diabetes, could consequently be considered to improve cardiovascular outcomes. Furthermore, all PPARs modulate angiogenesis, a critical process in cardiovascular pathologies. Although many research studies suggested beneficial effects of PPAR activation in the setting of cardiovascular disease (reviewed in [4,5]), they only have limited effects on cardiovascular outcomes in clinical studies (reviewed in [6,7,8]). In this review, we intend to summarize the angiogenic functions of PPARs with respect to their implications in cardiovascular pathology and analyze their utility in pharmacological therapies of cardiac disease states.

## 2. Peroxisome Proliferator Activated Receptors (PPARs)

**Figure 1:**
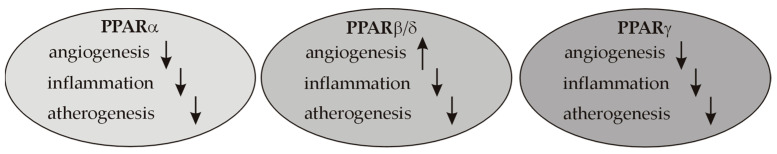
Simplified scheme of vascular PPAR actions with relevance for cardiovascular disease. ↑ indicates increase; ↓ indicates decrease.

The endothelial expression of all PPARs was demonstrated 20 years ago [9]. Although some conflicting results have been reported, in general, the activation of PPARα and PPARγ is linked to antiangiogenic effects, while PPARβ/δ activators favor angiogenesis (reviewed in [8]). Vascular aging is associated with structural and functional changes to the vasculature and represents an important risk factor for cardiovascular disease. Vascular aging leads to increased oxidative stress, disturbing metabolic and hemodynamic mechanisms (reviewed in [10,11]). Although reduced telomere length resulting in DNA damage, impaired replicative capacity of cells, and upregulated vascular cell senescence is clearly a hallmark of cardiovascular aging [12], it has been shown recently that, in most organs, an age-dependent diminution of endothelial cells and pericytes precedes vascular senescence. The implication is that the vascular cell loss drives senescence. The molecular analysis of endothelial cells from different tissues with age-induced vascular cell loss compared to endothelial cells from organs without age-related vascular cell disappearance revealed inflammatory processes as a common nominator in this process [13,14]. As all PPARs are considered to suppress vascular inflammation and atherogenesis (Figure 1) and be strongly involved in vascular metabolic and hemodynamic mechanisms, they are attractive candidates in the therapy of cardiovascular diseases.

### 2.1. Peroxisome Proliferator Activated Receptor Alpha (PPARα)

PPARα activation in the vasculature represses inflammatory responses, which involves the inhibition of the cytokine-induced vascular cell adhesion molecule-1 (VCAM-1), thus limiting the recruitment of inflammatory cells to the activated endothelium. This is, in part, achieved through the inhibition of the nuclear factor κB (NFκB) activation [15,16]. PPARα negative interference with NF-κB and activator protein (AP)-1 transcriptional activities [17] is further implicated in cyclooxygenase-2 (COX-2), interleukin-6, prostaglandin [18], monocytic tissue factor (TF) [19], and endothelin-1 (ET-1) [20] repression. PPARα activators enhance endothelial nitric oxide (NO) synthase expression and NO release [21,22]. Thus, the regulation of the vascular tone by stimulating NO-dependent vasodilation and inhibiting ET-1-induced vasoconstriction further contributes to the vasoprotective effects of PPARα activation.

PPARα antiangiogenic functions are mediated through decreased Akt (protein kinase B) activation, COX-2 [23], prostaglandin E (PGE)(2) [24], and matrix metallopeptidase (MMP) 9 [25], as well as vascular endothelial growth factor (VEGF) expression and an increase of antiangiogenic endostatin and thrombospondin (TSP)-1 [26]. A cardiomyocyte-specific overexpression of PPARα in genetic mouse models recapitulates a diabetic heart phenotype with cardiomyopathy due to lipid accumulation in cardiomyocytes. The expression of genes involved in cardiac fatty acid uptake and oxidation pathways increased while those of genes involved in glucose transport and utilization decreased, which consequently led to higher myocardial fatty acid oxidation rates and lower glucose uptake and oxidation [27,28]. Most animal studies applying ischemia/reperfusion or myocardial infarction procedures reported beneficial effects of PPARα agonists reflected by reduced infarct sizes and improved cardiac function (reviewed in [5]). In humans, the Fenofibrate Intervention and Event Lowering in Diabetes (FIELD) study randomized 9795 patients with Type 2 diabetes mellitus and dyslipidemia to fenofibrate or a placebo. Fenofibrate did not significantly reduce the composite primary end point of nonfatal myocardial infarction and coronary heart disease mortality. However, it reduced total cardiovascular events, mainly due to fewer nonfatal myocardial infarctions and revascularizations, as a 21% reduction of coronary vascularization was observed in the fenofibrate-treated subjects [29]. Clinical trials using PPARα agonists are summarized in Table 1. The ACCORD (action to control cardiovascular risk in diabetes) trial combined statin (simvastatin) therapy with fenofibrate in patients with Type 2 diabetes mellitus. The combination of the PPARα agonist fenofibrate and simvastatin did not reduce the overall rate of fatal cardiovascular events, nonfatal myocardial infarction, or nonfatal stroke as compared with simvastatin alone [30]. A newer pharmacogenetic analysis of the ACCORD-Lipid trial identified a common variant at the PPARA locus (rs6008845, C/T). T/T homozygous patients for this variant experienced a 51% reduction in major cardiovascular events in response to fibrate treatment, even in the absence of atherogenic dyslipidemia. Also, African Americans from the ACCORD cohort and from external cohorts (ACCORD-BP, ORIGIN, and TRIUMPH) showed a significant benefit from fibrate treatment for the T/T variant [31]. Interestingly, this variant was associated with lower levels of circulating CCL11, which is a proinflammatory and atherogenic cytokine [31]. Fibrate pharmacogenomics have been reviewed in detail by House & Motsinger-Reif [32]. The identification of PPARA common variants, which are associated with a clear cardiovascular benefit from fibrate treatment, justifies additional clinical studies. Unfortunately, although sequencing technologies are more easily available in recent years, it is still not common clinical practice to prescribe widely used drugs like fibrates on the basis of identified genetic variants.

The Helsinki heart study randomized 4180 men with primary dyslipidemia to the PPARα agonist gemfibrozil or a placebo. Gemfibrozil lowered the incidence of coronary heart disease by 34% [33]. Similarly, in the Veterans Affairs High-Density Lipoprotein Cholesterol Intervention Trial, which randomized 2531 men, Gemfibrozil therapy resulted in a significant reduction (22%) in the risk of major cardiovascular events in patients with coronary disease whose primary lipid abnormality was a low HDL cholesterol level [34,35]. The recent Pemafibrate to Reduce Cardiovascular Outcomes by Reducing Triglycerides in Patients with Diabetes (*PROMINENT*) trial for Pemafibrate - a highly active and selective PPARα agonist - in diabetic patients [36] was stopped in April 2022. Reviewing the results of an interim analysis, the Data Safety Monitoring Board (DSMB) concluded that it was unlikely that the primary endpoints would be met. The absolute treatment effects of fibrates in the primary prevention of cardiovascular events appear to be modest, as fibrates improve cardiovascular outcomes only in specific high-risk populations (fenofibrate in diabetic patients with metabolic syndrome, as well as gemfibrozil in patients with dyslipidemia).

### 2.2. Peroxisome Proliferator Activated Receptor Beta/Delta (PPARβ/δ)

Similar to PPARα and PPARγ, PPARβ/δ activation inhibits major vascular inflammatory responses through the inhibition of NFκB [37], VCAM-1 [37,38,39], E-selectin [38,39], and the intercellular adhesion molecule (ICAM)-1 [38,40]. In addition, PPARβ/δ controls inflammation through ligand-dependent interaction with B-cell lymphoma (BCL)-6. In the unliganded state, PPARβ/δ binds to the transcriptional repressor BCL-6. Upon ligand activation of PPARβ/δ, BCL-6 is released and can suppress pro-inflammatory pathways responsible for atherosclerosis formation [41]. PPARβ/δ further induces transforming growth factor (TGF) β1, leading to the induction of the antithrombotic plasminogen activator inhibitor-1 and the decrease of the proinflammatory chemokine monocyte chemoattractant protein (MCP)-1 [42]. PPARβ/δ also protects vascular cells from tissue-damaging oxidant stress, which itself would enhance inflammatory reactions. PPARβ/δ agonists induce heme oxygenase-1 expression, rendering endothelial cells in culture resistant to cellular stress induced by hydrogen peroxide or leptin [43]. PPARβ/δ further increases 14-3- 3α protein [44], superoxide dismutase-1, and catalase and thioredoxin expression [38]. The expression of these antioxidant genes represses reactive oxygen species (ROS) production in vascular cells, thus orchestrating a powerful antioxidant response.

In strong contrast to PPARα and PPARγ, the activation of PPARβ/δ induces pro-angiogenic responses in vitro and in vivo [45,46,47,48,49,50,51], reviewed in [8]. Pro-angiogenic targets of PPARβ/δ include VEGF [45], Cl−intracellular channel protein (CLIC) 4 [52], which is required for endothelial tube formation [53], calcineurin, which leads to the activation of nuclear factor of activated T cells (NFAT) c3 and the induction of pro-angiogenic hypoxia-inducible factor (HIF)-1 [48], as well as platelet-derived growth factor receptor (PDGFR) β and tyrosinkinase KIT (c-Kit) [50].

PPARβ/δ is the dominant PPAR subtype expressed in cardiac tissue [54]. The cardiomyocyte-specific overexpression of PPARβ/δ increases FAO and glycolysis. The increased capacity for myocardial glucose utilization was found to be beneficial in the setting of myocardial injury due to ischemia/reperfusion [28]. The cardiomyocyte-specific overexpression of PPARβ/δ further induced cell cycle progression of cardiomyocytes in myocardial infarction models, resulting in improved cardiac function [55]. The PPARβ/δ agonist GW610742X did not change left ventricular functional parameters or infarct sizes but normalized cardiac substrate metabolism and reduced right ventricular hypertrophy and pulmonary congestion after experimental myocardial infarction in rats [56]. Indirect cardioprotective functions of PPARβ/δ have been proposed in a study investigating the beneficial effects of remote ischemic preconditioning (rIPC) for cardiac protection after myocardial infarction. The protective effects of rIPC were mediated via the phosphoinositide 3-kinase (PI3K)/Akt/glycogen synthase kinase 3β (GSK3β) signaling pathway, which induced the nuclear accumulation of β-catenin and upregulation of its downstream targets E-cadherin and PPARβ/δ, which are involved in cell survival. Although the authors observed reduced apoptosis, infarct sizes, and improved functional recovery in mice, the exact impact of PPARβ/δ is not clear [57]. However, vascularization after myocardial infarction and the pro-angiogenic effects of PPARβ/δ on the functional outcome have not been investigated in any of these experimental studies. Our group used mice with inducible vascular-specific overexpression of PPARβ/δ, in which we found a rapid increase of cardiac vascularization accompanied by a fast onset of myocardial hypertrophy and impaired cardiac function. In addition, in the setting of myocardial infarction, PPARβ/δ vessel-specific overexpression increased capillary densities but failed to improve the outcome, as reflected by bigger infarct sizes, increased fibrosis, and significantly impaired echocardiographic parameters [49]. In line with our findings, treatment with the PPARβ/δ agonist GW610742 has been reported to increase vessel densities and fibrosis after myocardial infarction in rats without having functional benefits [58]. It seems that the specific, unbalanced activation of PPARβ/δ in only the vasculature is not sufficient to protect against the cardiomyocyte damage in chronic ischemic heart disease. Consequently, for PPARβ/δ modulation in cardiovascular disease, clinical trials are rare. The effects of the PPARβ/δ agonist GW501516 were investigated in pre-clinical trials to treat metabolic syndrome and diabetes at the beginning of 2000. Trials were stopped in 2007 due to the appearance of multiple cancers in mice and rats [3]. Not surprisingly, no clinical trials for the use of selective PPARβ/δ agonists in cardiovascular disease exist. The angiotensin II receptor blocker telmisartan targets PPARβ/δ [59] as well as PPARγ [60,61]. Two clinical trials for telmisartan have been completed: TRANSCEND (Telmisartan Randomized Assessment Study in ACE-Intolerant Subjects with Cardiovascular Disease) and ONTARGET (Ongoing Telmisartan Alone and in Combination with Ramipiril Global Endpoint Trial). In terms of primary and secondary outcomes, no significant differences were observed between the groups, with the exception of female patients who showed a 20% overall risk reduction for myocardial infarction [62]. If this limited beneficial effect of telmisartan can be attributed to angiotensin II receptor blockade, PPARγ activation, or PPARβ/δ activation is difficult to conclude.

### 2.3. Peroxisome Proliferator Activated Receptor Gamma (PPARγ)

Like the other PPARs, PPARγ activation inhibits tissue and endothelial cell inflammatory responses. PPARγ agonists decrease expression of the adhesion molecule ICAM-1 by suppressing NF-κB/DNA binding activity [63]. They inhibit pro-inflammatory molecules such as MMP-9 [64], chemokines monocyte chemoattractant protein (MCP)-1 [65], IFN-inducible protein of 10 kDa (IP-10), monokine induced by IFN-gamma (Mig), and IFN-inducible T-cell alpha-chemoattractant (I-TAC) [66], as well as cytokine IL-8 [67] and vasoactive mediators inducible nitric oxide synthase (iNOS) [68] and ET-1 [17]).

The activation of PPARγ has mostly been demonstrated to inhibit angiogenesis [9,69]. PPARγ agonists do not only suppress transcription of VEGF [70], but also of the vascular endothelial growth factor receptor (VEGFR) 2 [71]. Antiangiogenic effects of PPARγ activation are further attributed to a pro-apoptotic mechanism due to PPARγ-mediated increased NO production [72], simultaneous activation of the p38 mitogen activated protein kinase (MAPK) pathway and inhibition of phosphorylation of p42/44 MAPK [73], and the suppression of protein kinase C (PKC)α -and C-AMP Response Element-binding protein (CREB)-mediated COX-2 expression in endothelial cells [74]. However, some in vivo studies establish angiogenic effects of PPARγ activation. In diabetic mice, blood flow recovery was impaired after hindlimb ischemia compared to nondiabetic controls. In diabetic mice, treatment with PPARγ agonists partially restored blood flow recovery and increased capillary density in ischemic hindlimbs [75,76]. Rosiglitazone increased angiogenesis, reduced apoptosis, improved functional recovery, and diminished the lesion size in animals with focal cerebral ischemia [77]. Pro-angiogenic effects of PPARγ agonist treatment in ischemic tissues are NO-dependent and related to endothelial nitric oxide synthase (eNOS) upregulation [76]. Although these studies seem to contradict the results of in vitro studies, they better take into account the interplay between different cell types, which might be responsible for the pro-angiogenic action of PPARγ activation in pathological in vivo situations. The cardiomyocyte-specific knockout of PPARγ induced cardiac hypertrophy, probably through the lack of inhibition of NF-κB [78], whereas the overexpression of PPARγ in cardiomyocytes resulted in cardiac myopathy characterized by increased lipid and glycogen storage and cardiac dysfunction [79]. Interestingly, the knockout of PPARγ in myeloid cells worsened the outcome after experimental myocardial infarction in mice, likely due to increased oxidative stress and cardiac inflammation [80]. Given the favorable anti-inflammatory and antiatherogenic effects of PPARγ activation in atherosclerosis, PPARγ agonists might lower the risk of cardiovascular disease. Accordingly, experimental animal studies using myocardial ischemia/reperfusion experiments all reported beneficial aspects with PPARγ activators (reviewed in [5]). The first small clinical trials showed some benefits of pioglitazone mainly concerning the amelioration of atherosclerotic inflammation [81,82,83,84]. Clinical trials using PPARγ agonists are summarized in Table 2. In 2005, the large PROspective pioglitAzone Clinical Trial In macroVascular Events (PROactive) trial randomized 5238 diabetic patients with macrovascular cardiovascular disease to pioglitazone or a placebo. The primary end points were all-cause mortality, nonfatal myocardial infarction, strokes, acute coronary syndromes, endovascular or surgical intervention in the coronary or leg arteries, and amputation above the ankle. No reduction in primary end points with pioglitazone could be demonstrated. However, pioglitazone reduced a composite secondary end point of all-cause mortality, nonfatal myocardial infarction, and strokes [85,86,87]. Later, in 2008, the PERISCOPE (Pioglitazone Effect on Regression of Intravascular Sonographic Coronary Obstruction Prospective Evaluation) trial compared the effects of pioglitazone and glimepiride (an antidiabetic Insulin secretagogue) in 543 patients with diabetes mellitus and coronary disease. The primary outcome was a change in the percentage of the atheroma volume from the baseline. After 18 months of follow-up, pioglitazone effectively reduced the progression of coronary atherosclerosis compared with glimepiride. The incidence of cardiovascular death and nonfatal myocardial infarction did not differ between the groups [88]. The more recent IRIS (Insulin Resistance Intervention After Stroke) trial evaluated the effectiveness of pioglitazone in patients with insulin resistance and a recent event of a stroke or a transient ischemic attack. The primary outcome was a fatal or nonfatal stroke, or a myocardial infarction. After five years, in 11.8% of the placebo group, a primary outcome event occurred. This was diminished to 9% in the pioglitazone cohort. Pioglitazone therapy was associated with higher risks of weight gain, edema, and fractures. Incident bladder cancer occurred in 12 patients in the pioglitazone group and in eight in the placebo group [89]. Thus, although pioglitazone might improve cardiovascular outcomes, there are major noncardiovascular safety concerns, such as an increased risk of bladder cancer (reviewed in [90]). In 2006, rosiglitazone was investigated in patients with impaired glucose tolerance. In the DREAM (Diabetes Reduction Assessment with Ramipiril and Rosiglitazone Medication) trial, 5962 patients were randomized to ramipiril (an angiotensin-converting enzyme inhibitor), rosiglitazone, ramipiril and rosiglitazone, and a placebo. The composite primary endpoint after three years was an incidence of diabetes, which was significantly lower with rosiglitazone. On the other hand, an increase in cardiovascular vents and congestive heart failure was observed in the patients receiving rosiglitazone [91]. In the ADOPT (A Diabetes Outcome Progression) trial, 4360 patients with diabetes were randomized to rosiglitazone, metformin, or glyburide. The primary outcome was the time to reach monotherapy failure, which was significantly reduced with rosiglitazone compared to the other two antidiabetic drugs [92]. In a meta-analysis study from 2007, 42 clinical trials were evaluated concerning the effects of rosiglitazone on the risk of myocardial infarction and death from cardiovascular causes, including the ADOPT and DREAM trials. The authors could conclude that rosiglitazone was associated with a significant increase in the risk of myocardial infarction and an increase in the risk of death from cardiovascular causes [93]. In the already ongoing RECORD (Rosiglitazone Evaluated for Cardiac Outcomes and Regulation of Glycaemia in Diabetes) trial, which assessed the addition of rosiglitazone to either metformin or sulfonylurea compared with the combination of the two in 4447 patients with diabetes mellitus, the addition of rosiglitazone to glucose-lowering therapy in patients with diabetes increased the risk of heart failure. Although the data were inconclusive about possible effects on myocardial infarction, rosiglitazone did not increase the risk of overall cardiovascular morbidity or mortality compared with standard glucose-lowering drugs [94]. A U.S. Food and Drug Administration (FDA) advisory meeting concluded that rosiglitazone carried considerable cardiovascular risks (including myocardial infarction risks [95]), leading to withdrawal in Europe and strong restrictions in the U.S.A. 

As for PPARα, also PPARγ variants modulate the risk for diabetes and cardiovascular events. The PPARG Pro12Ala allele was associated with a reduced risk of myocardial infarction in a Type 2 diabetic population, while the T allele of the C1431T polymorphism was associated with increased all-cause mortality [96]. A study using a community-based cohort from Maryland did not find associations between PPAR polymorphisms and the risk of cardiovascular morbidity and mortality [97].

Pan PPAR agonists (Table 3) were designed in order to treat dyslipidemia and diabetes with a single drug (reviewed in [98]). Bezafibrate is the first and most studied PPAR pan agonist. In 1998, the SENDCAP (St. Mary’s, Ealing, Northwick Park Diabetes Cardiovascular Disease Prevention) study randomized 164 diabetes patients to bezafibrate or a placebo. Bezafibrate improved dyslipidemia but had no effect on the progress of arterial disease. Nevertheless, it significantly decreased the incidence of definite coronary heart disease events over 3 years [99]. Later, the BIP (Bezafibrate infarction Prevention) trial randomized 3090 patients with previous myocardial infarction or stable angina and hyperlipidemia to bezafibrate or a placebo. The composite endpoints were myocardial infarction or death. Bezafibrate effectively lowered triglycerides, but only an overall, not significant trend to reduce the incidence of primary end points was observed [100].

The experimental PPAR pan agonists muraglitazar, ragaglitazar, and tesaglitazar were initially promising, but serious side effects were observed during testing. Clinical trials using pan-or dual (α/γ) PPAR agonists for cardiovascular disease are summarized in Table 3. The meta-analysis of five clinical trials involving 2374 patients exposed to muraglitazar and 1351 patients exposed to comparator agents, of which 823 received pioglitazone and 528 a placebo, revealed that muraglitazar was associated with an excess incidence of death and major adverse cardiovascular events [101]. Clinical trials for ragaglitazar have been stopped after the discovery of its carcinogenic effects on rodent bladders [102]. The clinical development of tesaglitazar has been discontinued due to its serum creatinine increasing the effect in diabetic patients [103]. Later trials, starting in 2006 with the dual PPARα/γ agonist aleglitazar (Synchrony, AleCardio, and Aleprevent randomized clinical trials), showed no significant improvement of cardiovascular disease but multiple negative side effects such as oedema, hemodilution, weight gain in the Synchrony trial [104], renal dysfunction and heart failure in the AleCardio trial [105]. The Aleprevent study had to be halted prematurely after the randomization of 1999 patients, although the initially intended sample size was 19,000 patients, due to hypoglycemia, edema, and adverse muscular events [106]. Given these unfavorable results, the clinical development of dual-PPAR agonist for cardiovascular disease has been stopped. Although glitazars improved metabolic parameters, they induced cardiac failure, which is probably due to glucolipotoxicity through the combined increase of PPARγ-driven insulin sensitization and glucose uptake in the setting of higher PPARα-induced activation of fatty acid uptake and oxidation. Unfortunately, the impact of a “doubled” inhibition of angiogenesis through simultaneous activation of PPARα and γ has never been investigated, but the lack of severe cardiovascular side effects in the pan agonist trials with bezafibrate could indicate that a balanced angiogenic response by activation of pro-angiogenic PPARβ/δ and antiangiogenic PPARα and γ could avoid major cardiovascular adverse effects.

**Table 3:** Summary of Clinical Trials with pan-or dual (α/γ) PPAR agonists for Cardiovascular Disease.

Study Name/Drug	Condition	Primary Endpoints	Outcome	Trial Identifier/Reference
St. Mary’s, Ealing, Northwick Park Diabetes Cardiovascular Disease Prevention (SENDCAP) study Bezafibrate	164 diabetic patients	Alteration of cardiovascular outcomes	No effect on the progress of ultrasonically measured arterial disease	[99]
Bezafibrate infarction Prevention (BIP) trial Bezafibrate	3090 patients with coronary artery disease	Myocardial infarction, sudden death	No reduction of myocardial infarction or death	[100]
Metanalysis of five clinical trials with Muraglitazar in Diabetic Patients Muraglitazar	3725 diabetic patients	Major adverse cardiovascular events, death	Excess incidence of death, myocardial infarction, congestive heart failure	[101]
Tesaglitazar vs. Placebo in Combination With Insulin (GALLANT9) trial Tesaglitazar	370 diabetic patients	Absolute change from baseline in glycosylated hemoglobin A1c (HbA1c)	Reduction of HbA1c), significant increase in serum creatinine	NCT00242372 [103]
A Study of Aleglitazar in Patients With Type 2 Diabetes (SYNCHRONY) trial	332 diabetic patients	Absolute change from baseline in glycosylated hemoglobin A1c (HbA1c)	Reduction of HbA1c), oedema, hemodilution, and weight gain	NCT00388518 [104]
Aleglitazar to Reduce Cardiovascular Risk in (CHD) Patients With a Recent Acute Coronary Syndrome (ACS) Event and Type 2 Diabetes Mellitus (T2D) (AleCardio) trial	7226 diabetic patients with myocardial infarction or unstable angina	Effect on cardiovascular death, nonfatal myocardial infarction, and nonfatal stroke	No reduction in the risk of cardiovascular outcomes	NCT01042769 [105]
Aleglitazar To Reduce Cardiovascular Risk In Patients With Stable Cardiovascular Disease And Glucose Abnormalities	1999 diabetic patients with cardiovascular disease	Reduction of cardiovascular morbidity and mortality	No reduction in the risk of cardiovascular outcomes	EudraCT Number: 2012-000671-16 [106]

## 3. Cardiovascular Disease Therapies Involving PPARs

To not completely dismiss the utility of PPARs in cardiovascular disease therapy, it is important to note that many conventional pharmacological therapies (Aspirin, Statins, Angiotensin-converting enzyme [ACE] inhibitors, and Angiotensin receptor blockers [ARBs]) for primary or secondary prevention of myocardial infarction often affect PPARs. Therefore, we include a brief review regarding their potential PPAR-related effects on cardiovascular pathophysiology.

### 3.1. Aspirin

Aspirin is one of the most frequently used drugs worldwide and is considered to be effective in the prevention of cardiovascular disease, mainly through its antithrombotic effects (reviewed in [107]). Additionally, it reduces cancer risk and mortality (reviewed in [108]). PPARα has recently been identified as a receptor for aspirin in the neuronal system [109]. Aspirin exerts neuroprotective functions through the stimulation of neurotrophin production, mediated through its binding to PPARα [110]. Neurotrophins are involved in the maintenance of cardiometabolic homeostasis and cardioprotection (reviewed in [111]), which might represent one aspect of the cardiovascular benefit of aspirin therapy mediated through PPARα. For aspirin, mainly antiangiogenic effects have been reported: it suppressed collateral artery growth in animal studies, possibly through cyclooxygenase (COX) inhibition [112]. Interestingly, antiangiogenic effects of PPARα activation have also been attributed to COX inhibition [23]. However, antiangiogenic effects of aspirin can’t be solely attributed to COX inhibition [113]. Aspirin reduces the production of pro-angiogenic 11-hydroxyeicosatetraenoic acid (HETE) and 15(S)-HETE [114], whereas PPARα ligands reduce the production of AA-derived epoxyeicosatrienoic acids (EETs) and increase their pro-angiogenic hydroxyl product, 11-HETE [115], possibly reflecting a balanced angiogenic response in the setting of cardiovascular and acute ischemic disease. In a small clinical study with patients with myocardial ischemia undergoing coronary artery bypass graft operation, aspirin significantly reduced VEGF release [116], an antiangiogenic mechanism also assigned to PPARα and γ activation (reviewed in [8]). It would be interesting to determine to what extent antiangiogenic effects of aspirin are mediated by PPAR activation.

### 3.2. Statins

Statins are frequently prescribed medications in the management of high cholesterol and associated cardiovascular complications. They lower cholesterol through inhibition of 3-hydroxy-3-methylglutaryl coenzyme A (HMG-CoA)-reductase [117]. Statin use is associated with a significantly reduced risk of all-cause mortality in cohorts with cardiovascular and noncardiovascular disease, including cancer [118]. Not only antiatherogenic and anti-inflammatory actions of statins have been associated with the activation of PPARα and γ ([119,120,121,122], but also cardioprotective statin effects, such as the inhibition of cardiac hypertrophy and fibrosis, can be attributed to PPARα and γ modulation [123,124,125]. Furthermore, statins have the potential to lower persistent elevated blood pressure levels through mechanisms implying PPAR activation [126]. In addition, the inhibition of platelet aggregation by statins is mediated through PPARs [127], which additionally contributes to vascular defensive effects of statins. Angiogenesis is stimulated at low (nanomolar) statin concentrations and inhibited by high (micromolar) doses [128,129,130]. Mechanisms of the pro-angiogenic effects of statins include activation of the (PI3K)/Akt pathway [130], an increase of VEGF, IL-8, Angiopoietin 1 and 2, eNOS, and heme oxygenase (HO)-1 expression [131]. This correlates quite well with the pro-angiogenic mechanisms of PPARβ/δ [132] and might indicate that angiogenic effects of statins are partially mediated by PPARs. This notion is further supported by the finding that statins induce transcriptional activation of PPARα/retinoid X receptor (RXR)α, PPARβ/δ/RXRα, and PPARγ2/RXRα [133].

### 3.3. Angiotensin-Converting Enzyme (ACE) Inhibitors

ACE inhibitors are commonly used in patients with hypertension, heart failure, coronary artery disease, diabetes, and chronic renal disease. They inhibit the activity of angiotensin-converting enzymes, an important component of the renin–angiotensin system that converts angiotensin I to angiotensin II (AngII). AngII promotes vascular inflammation through NF-κB-mediated induction of pro-inflammatory genes and downregulation of PPARα and γ [134]. ACE inhibitors upregulate PPAR expression [135,136]. Like ACE inhibitors, PPARs inhibit AngII synthesis [137]. Therefore, they also contribute to antiatherogenic and anti-inflammatory effects. In chronic daunorubicin-induced cardiomyopathy, ACE inhibition reestablished normal PPARβ/δ and γ levels, along with a reduction of NADPH (nicotinamide adenine dinucleotide phosphate) oxidase. However, a possible causal link between these effects has not been investigated [138]. Ang II enhances angiogenesis through angiotensin Type 1 receptor activation (AT1R) that involves VEGF/eNOS-dependent pathways [139]. Both subtypes of receptors, AT1R and AT2R, are implicated in angiogenic processes [140,141]. ACE inhibitors do not solely inhibit the activity of pro-angiogenic AngII, but also the degradation of bradykinin, which mediates pro-angiogenic functions through binding to its receptors B1 and B2 [142]. Not surprisingly, the inhibition of ACE, affecting AngII and bradykinin degradation, has different impacts on angiogenesis. ACE inhibitors were reported to promote angiogenesis in hindlimb ischemia models [143,144,145] and to promote myocardial capillary formation [146,147,148,149]. An analysis of ACE inhibitor effects on retinal and hindlimb angiogenesis in diabetic mice revealed an induction of neovascularization in the hindlimbs through the activation of bradykinin signaling, and a reduction of retinal vascularization through the inhibition of AngII [150]. In addition, in a rabbit model of VEGF-induced corneal vascularization, ACE inhibition significantly reduced angiogenesis [151]. Similarly, in exercise-induced angiogenesis, ACE inhibitors reduced VEGF expression and neovascularization [152]. Importantly, although ACE inhibitors have some pro-angiogenic effects, they reduce tumor angiogenesis [153,154,155]. A meta-analysis of randomized trials with ACE inhibitors did not find any impact on the occurrence of cancer or cancer death [156], and ACE inhibition is nowadays considered as a possible complementary treatment option for cancer patients (reviewed in [157].

### 3.4. Angiotensin Receptor Blockers (ARBs)

ARBs were developed to overcome some deficiencies of ACE inhibitors: competitive inhibition of ACE results in a reactive increase in renin and angiotensin I levels, which may overcome the blockade effect. ACE is a relatively nonspecific enzyme that has substrates in addition to angiotensin I, including bradykinin. Therefore, the inhibition of ACE may result in accumulation; the production of AngII can occur through non-ACE pathways. Therefore, ARBs may offer more complete AngII inhibition by interacting selectively with the angiotensin Type 1 receptor site (reviewed in [158]). Their indications are the same as those for ACE inhibitors. The ARBs irbesartan and, mostly, telmisartan induce PPARγ activity [61,159] and were, therefore, considered as highly beneficial in treating cardiovascular, metabolic, and inflammatory diseases in insulin-resistant and euglycemic states reviewed in [160]. Interestingly, telmisartan provides a triple inhibition of angiotensin II function: AT1R blockade, downregulation of AT1R expression [161], and ACE inhibition [162]. The reduced expression of AT1R and vascular ACE inhibition were attributed to PPARγ activation [161,162]. Telmisartan further elicits anti-inflammatory and antiatherosclerotic effects through the activation of PPARγ, involving reduced activity of the pro-inflammatory transcription factors NF-kB and early-growth response protein (Egr)-1 [163], promotes the proliferation of endothelial progenitor cells [164], and improves microvascular dysfunction during myocardial ischemia/reperfusion injury [165] via PPARγ dependent pathways. Telmisartan was approved by the FDA for the treatment of hypertension in November 1998. In 2009, telmisartan was the first ARB to be granted FDA approval for the reduction of cardiovascular risk in high-risk patients unable to take ACE inhibitors. In addition to telmisartan, other ARBs have been described to activate PPARγ [166,167,168,169,170,171,172], but also PPARα [173,174,175,176,177] and PPARβ/δ [175,178,179]. PPARβ/δ activation by telmisartan has been reported to reduce cardiac fibrosis in diabetic animals [179], inhibit inflammation of endothelial cells [180], ameliorate insulin resistance in skeletal muscle [178], prevent obesity [181], exert neuroprotective effects [182], and even improve symptoms of stress-induced depression [183].

In general, ARBs have antiangiogenic effects. They were shown to inhibit neoangiogenesis in ischemia hindlimb models [184], impair VEGF-induced revascularization in cardiomyopathy [185] and diabetes [140], and impede hypoxia-induced coronary angiogenesis [186]. The combined blockade of angiotensin Type 1 receptor and the activation of PPARγ by telmisartan inhibited vascularization in several models of angiogenesis [187]. However, PPARγ-independent mechanisms in the inhibition of vascular cell proliferation by telmisartan, involving repression of Akt, have been postulated [188]. 

Importantly, ARBs inhibit tumor angiogenesis and tumor growth, involving the repression of VEGF [189,190]. Telmisartan was shown to exert antitumor effects in vitro through PPARγ activation and subsequent downregulation of MMP9 and ICAM-1 [191,192].

Despite these encouraging experimental results, a first meta-analysis of randomized controlled trials in 2010 suggested that ARBs, mainly telmisartan, are associated with a modestly increased risk of new cancer diagnosis [193]. In a novel meta-analysis of the same year, covering 70 randomized controlled trials for antihypertensive drugs, the 5–10% relative increase in the risk of cancer or cancer-related death with the use of ARBs established in the former study was refuted [194]. Accordingly, a meta-analysis of 15 trials for different ARBs enrolling 138,769 patients did not find an excess of cancer incidence [195].

Recently, the design of tumor microenvironment-activated ARBs has been proposed to reprogram cancer associated fibroblasts to an immunosupportive state, diminishing immunosuppression and improving T lymphocyte activity in cancer [196].

## 4. Conclusions

In conclusion, the utility of PPAR agonists for the treatment of cardiovascular disease, beneficial modelling of angiogenesis, and myocardial infarction prevention is limited. Although they seem to reduce cardiovascular risk factors, they failed to improve cardiovascular outcomes in clinical trials, were only moderately effective in very selective subgroups of patients, or even produced severe side effects. The observed heterogenous effects of PPAR agonists in clinical studies highlight the importance of a strict post marketing surveillance. Established conventional cardiovascular medications often include PPAR-activating mechanisms, which might contribute to their beneficial low risk effects. Balanced indirect or coupled PPAR activation by these medications seems to be preferable to unique, unbalanced PPAR activation in the setting of cardiovascular diseases.

## Figures and Tables

**Table 1:** Summary of Clinical Trials with PPARα agonists for Cardiovascular Disease.

Study Name/Drug	Condition	Primary Endpoints	Outcome	Trial Identifier/Reference
Fenofibrate Intervention and Event Lowering in Diabetes (FIELD) study Fenofibrate	9795 patients with diabetes and dyslipidemia	Nonfatal myocardial infarction, coronary heart disease mortality	No significant reduction of coronary events	number ISRCTN 64783481 [29]
Action to control cardiovascular risk in diabetes (ACCORD) trial Simvastatin alone or in combination with Fenofibrate	5518 patients with Diabetes	Nonfatal myocardial infarction, nonfatal stroke, cardiovascular death	No reduction of nonfatal myocardial infarction, nonfatal stroke, cardiovascular death	NCT00000620 [30]
Helsinki Heart Study Gemfibrozil	4081 male patients with hyperlipidemia	Cardiac death, myocardial infarction	Reduction of coronary heart disease progression	[33]
Veterans Affairs High-Density Lipoprotein Cholesterol Intervention Trial Gemfibrozil	2531 male patients with dyslipidemia and coronary artery disease	Myocardial infarction, death from coronary artery disease	Reduction of major cardiovascular events	[34,35]
Pemafibrate to Reduce Cardiovascular Outcomes by Reducing Triglycerides in Patients with Diabetes (*PROMINENT*) trial Pemafibrate	10,544 patients with diabetes	Nonfatal myocardial infarction, nonfatal stroke, cardiovascular death	Stopped in 2022 due to futility	[36] NCT03071692

**Table 2:** Summary of Clinical Trials with PPARγ agonists for Cardiovascular Disease.

Study Name/Drug	Condition	Primary Endpoints	Outcome	Trial Identifier/Reference
PROspective pioglitAzone Clinical Trial In macroVascular Events (PROactive) trial Pioglitazone	5238 patients with diabetes and cardiovascular disease	Time to first occurrence of macrovascular events or death	No significant reduction of coronary events, higher incidence of heart failure	NCT00174993 [85,86]
Insulin Resistance Intervention After Stroke Trial (IRIS) trial Pioglitazone	3876 patients with insulin resistance and stroke or myocardial infarction	Recurrent fatal or nonfatal Stroke, or fatal or nonfatal myocardial infarction	Lower risk of stroke or myocardial infarction	NCT00091949 [89]
Diabetes Reduction Assessment with Ramipiril and Rosiglitazone Medication (DREAM) trial Rosiglitazone	5962 patients with impaired glucose tolerance	Incidence of diabetes	Lower incidence of diabetes, increase in cardiovascular events	NCT00095654 [91]
A Diabetes Outcome Progression (ADOPT) trial Rosiglitazone	4360 diabetic patients	Time to monotherapy failure	Time to monotherapy failure reduced	NCT00279045 [92]
Rosiglitazone Evaluated for Cardiac Outcomes and Regulation of Glycaemia in Diabetes (RECORD) trial Rosiglitazone	4447 diabetic patients	Cardiovascular events, long-term glycemic control	No reduction of overall cardiovascular morbidity or mortality	NCT00379769 [94]

## Data Availability

Not applicable.

## Abbreviations

ACE	Angiotensin Converting Enzyme Inhibitor
Akt	Protein Kinase B
AngII	Angiotensin II
ARB	Angiotensin Receptor Blocker
AP-1	Activator Protein-1
AT1R	Angiotensin Type 1 Receptor
BCL-6	B-cell lymphoma-6
c-Kit	Tyrosinkinase Kit
CLIC 4	Cl−intracellular channel protein 4
COX-2	Cyclooxygenase-2
CREB	C-AMP Response Element-binding protein
EGR	Early Growth Response Protein
eNOS	endothelial Nitric Oxide Synthase
ET-1	Endothelin-1
FAO	Fatty Acid Oxidation
FDA	Food and Drug Administration
GSK3β	glycogen synthase kinase 3β
HDL	High Density Lipoprotein
HETE	hydroxy eicosatetraenoic acid
HIF-1	Hypoxia Inducible Factor 1
HMG-CoA	3-hydroxy-3-methylglutaryl coenzyme A
HO	Heme Oxygenase
ICAM-1	Intercellular Adhesion Molecule-1
IP-10	IFN-inducible protein of 10 kDa
I-TAC	IFN-inducible T-cell alpha-chemoattractant
MAPK	Mitogen Activated Protein Kinase
MCP	Monocyte Chemoattractant Protein
MMP	Matrix Metallopeptidase
Mig	monokine induced by IFN-gamma
NADPH	nicotinamide adenine dinucleotide phosphate
NFκB	nuclear factor κB
NO	Nitric Oxide
NFAT	nuclear factor of activated T cells
PDGFR	Platelet-derived Growth Factor Receptor
PI3K	phosphoinositide 3-kinase
PPAR	Peroxisome proliferator activated receptor
PGE	Prostaglandin E
PKC	Protein Kinase C
rIPC	Remote Ischemic Preconditioning
RXR	Retinoid X Receptor
TSP	Thrombospondin
TF	Tissue Factor
TGF	Transforming Growth Factor
VCAM-1	Vascular Cell Adhesion Molecule 1
VEGF	Vascular Endothelial Growth Factor
VEGFR	Vascular Endothelial Growth Factor Receptor
